# Determination of optimal mining width for coal mining under the slope by of using numerical simulation

**DOI:** 10.1038/s41598-024-51624-4

**Published:** 2024-01-11

**Authors:** Ze Zhou, Jinlian Zhou, Long Lai, Mengtang Xu, Youlin Xu

**Affiliations:** 1 Institute of Mining Engineering, Guizhou Institute of Technology, Guiyang, China; 2 Huopu Coal Mine, Guizhou Pangjiang Refined Coal Co. Ltd., Guiyang, China; 3 Guizhou Energy Administration, Guiyang, China

**Keywords:** Civil engineering, Applied mathematics

## Abstract

The stability of slope is critical important topic in rock mass engineering. In Panzhihua #7 Coal Mine, the coal mining is occurred under the slope, to obtain the optimal mining width, 125 numerical simulations were conducted, and the corresponding FOS was calculated. From the analysis of the numerical simulation results, it can be found that FOS decrease and then increase with increasing of filling length, meanwhile, the FOS is minimum value when the filling length is within the ranges of 10 m and 20 m, which is under the toe of slope. Furthermore, the minimum FOS decrease with increasing of mining width. Considered the stability of the slope and mining economy, the mining width is set to 6m, and the numerical simulation results is applied to the engineering practice. To guarantee the safety of the slope, some monitoring points were distributed on the slope, the displacement from numerical simulation and engineering practice is quite close, indicating the numerical simulation results is reliable, and the mining width is reasonable.

## Introduction

Coal is the importance strategic resource of our country, and it plays a critical important role in the development of our national economy^1^. With the excavation of coal, more and more coal resources were discovered, and some coal was under the slope, during coal mining process, the stability of slope would be influenced, which would result in sliding of slope or collapse, thereof, the stability of slope during the coal mining process should be taken enough attention.

The many factors influence the stability of slope, such as geological background^2–4^, rock mass structure^5–8^, lithology^8–10^, topographic features^11–13^, hydrogeological condition^14–17^. To describe the stability of slope quanttitatively, different method was used. The finite element method is widely used and effective technique to assess the stability of the slope^18–22^, however, the finite element method can not solve the large deformation and displacement discontinuity problem. Based on the finite element method, the adaptive finite element method is proposed^23,24^, by using this technique, the seepage dynamics problems can be solved in short time, which indicated the technique has a fast convergence rate. It should be noted that the adaptive finite element method still can not solve the displacement discontinuity problem, thereof, the distinct element method (DEM) was proposed by Cundall^25–28^, by using this techniques, the displacement between the rock blocks and be easily simulated, moreover, nonlinear deformation and dynamic problems can be solved quickly, and the method is widely used for stability analysis of rock slope. Apart from the DEM, the discontinuous deformation analysis (DDA) proposed by Goodman^30–33^ is a newly numerical simulation method, in this method, discontinuity of deformation and time factor were considered, the static problem and dynamic problem can be easily and quickly solved. In engineering practice, the DEM and DDA would take a lot of time to solve a problem by comparison with the finite element method, considered the application of engineering practice, fast Lagrangion analysis of continue (FLAC)^34,35^ was proposed, the FLAC method can reflect the large deformation of rock mass and discontinuity characteristics, and the process can be implemented in a short time, thereof, the FLAC3D commercial software was widely applied in rock mass engineering practice. Apart from the method described above, there are many other effective way to estimate the stability of slope, such as, grey system theory^36^, cluster method^37^, expert systems^38^, neural network^39^, and so on. These method can provide effective ways to depict the stability of slope.

From analysis above, the present studies about the slope are mainly focused on the method to estimate the stability of slope, however, the related references about the slope stability under the coal excavation was less. Thereof, in this paper, the numerical simulation about the influence of underground of coal mining to the stability of slope was conducted, and the stability of slope when the mining width and filling length was analyzed, and the optimal mining width was propose, and the numerical simulation results were applied to the engineering practice, from the monitoring results from the numerical simulation and engineering practice, it can be found that the results from numerical simulation is close to the engineering practice, which indicated that the numerical simulation is reliable and workable. This study can provide numerical analysis basis for influence of coal mining to the stability of slope.

## Numerical simulation

### Model construction and numerical simulation process

**Figure 1:**
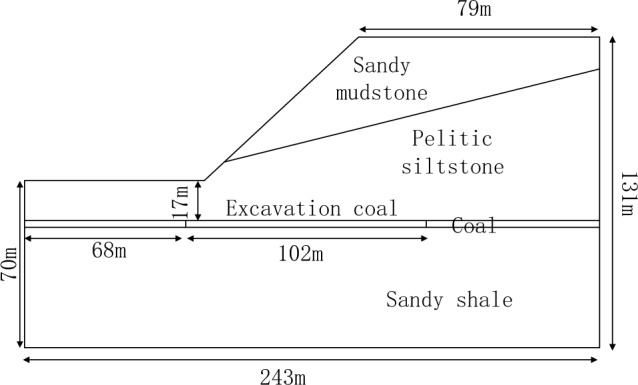
Geometry of the numerical simulation model.

In this paper, the Panzhihua #7 Coal Mine was taken as the numerical simulation example, as illustrated in Fig. 1, it can be observed that there 4 layers for the numerical simulation model, which are sandy mudstone, pelitic siltstone, coal and sandy shale. The angle of slope is 43°, and other geological structure geometry parameters of layer distribution can be seen in Fig. 1.

**Figure 2:**
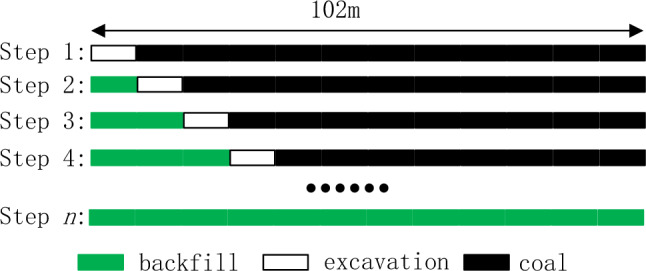
Coal excavation and backfill process.

In the coal mine, backfill mining method is used, and the gangue gypsum filling materials was used, by using this materials, the gangue generated during the coaling process was fully used and the waste was reduce. In the mining process, after the coal excavation, the backfill materials would filled in the goaf, for convenience of production, the excavation width is the same value during the mining process, which can be seen in the Fig. 2.

**Figure 3:**
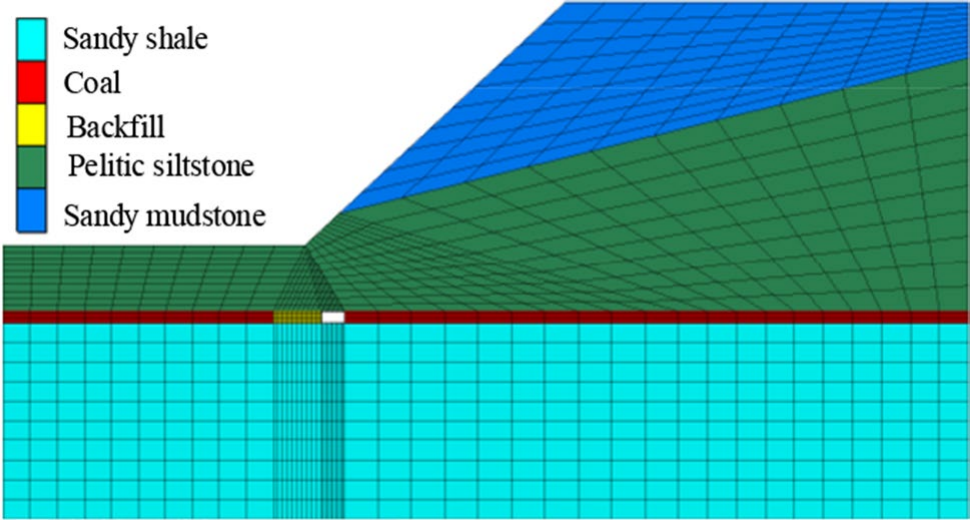
Numerical simulation model (Fill length is 12m, mining width is 6 m).

As shown in Fig. 2, during the coal mining and backfill process, the mining width is the same, and after the next area coal was excavated, the excavated area was filled with backfill materials. For the step 1, coal was excavated, and no backfill area, while for step 2, the 95% space of goaf was filled with backfill materials, and one area was excavated, until all coal was excavated and goaf was filled with backfill materials, the coal length is 102 m. Based on the geological structure parameters, mining and backfill process, the corresponding numerical simulation model was constructed, which is displayed in Fig. 3.

In the numerical simulation model, the Mohr–Coulomb model is used, and the corresponding mechanical parameters were listed in Table 1.

**Table 1:** Mechanical parameters of numerical simulation model.

Parameter	Density (kg/m^3^)	Bulk modulus (Pa)	Shear modulus (Pa)	Friction angle (^o^)	Cohesion (Pa)	Tension (Pa)
Sandy mudstone	2230	3.34e8	1.36e8	15	11.2e4	8.3e4
Pelitic siltstone	2450	4.92e8	3.09e8	17	12.5e4	9.4e4
Coal	1400	0.66e8	0.25e8	14	5.4e4	8.4e4
Sandy shale	2315	1.8e8	0.68e8	16	9.3e4	9.6e4
Backfill materials	2350	2.13e8	1.75e8	15	5.6e4	8.8e4

In the mining process, the mining width is larger, the mining process would be more economy, however, it would be decrease the stability of the slope, thereof, to determine the optimal mining width, the mining width with 5 m, 6 m, 7 m, 8 m, 9 m, 10 m, 11 m and 12 m were selected, the corresponding numerical simulation were conducted. Meanwhile the factor of safety of slope is used to describe the stability during the coal mining process, in the manuscript, the slope is unstable when the factor of safety is less than or equal to 1.

### Factor of safety calculation

The “strength reduction technique” is used to estimate the Factor of Safety (FOS) of slope in FLAC3D commercial software^34,35^. The “strength reduction technique” is typically applied in factor of safety calculations by progressively reducing the shear strength of the material to bring the slope to a state of limiting equilibrium. The method is commonly applied with the Mohr–Coulomb failure criterion^40^. In this case, the safety factor *F* is defined according to the equations:

1 $$\left\{ \begin{gathered} c^{{{\text{trial}}}} = \frac{c}{{F^{{{\text{trial}}}} }} \hfill \\ \phi^{{{\text{trial}}}} = \arctan \left( {\frac{\tan \phi }{{F^{{{\text{trial}}}} }}} \right) \hfill \\ \end{gathered} \right.$$

A series of simulations are made using trial values of factor $F^{{{\text{trial}}}}$ to reduce the cohesion $c$ and friction angle $\phi$, until slope failure occurs. (Note that if the slope is initially unstable, $c$ and $\phi$ will be increased until the limiting condition is found). Once technique to find the strength values that correspond to the onset of failure is to monotonically reduce (or increase) the strengths in small increments until failure state is found. Alternatively, in FLAC3D, a bracketing approach is used. With the technique, stable and unstable bracketing states are found first, and then the bracket between stable and unstable solution is progressively reduced until the difference between stable and unstable solutions falls below a specified tolerance.

The detection of the boundary between physical stability and instability is based on an objective criterion in FLAC3D that decides whether the system is in equilibrium or a state of continuing motion. Finer incremental changes that may affect the solution in an iterative solution scheme are not needed in a time-marching scheme and do not affect the solution. In order to determine the boundary between physical stability and instability, a set of completely separate runs is made with different strength-reduction factors. Each run is then checked to determine whether equilibrium or continuing plastic flow is reached. The point of failure can be found to any required accuracy (typically 1%) by successive bracketing of the strength-reduction factors. This process should not be confused with taking finer solution steps; the solution scheme is identical for each run of the set (whether it results in equilibrium or continuing motion).

### Numerical simulation results analysis

**Figure 4:**
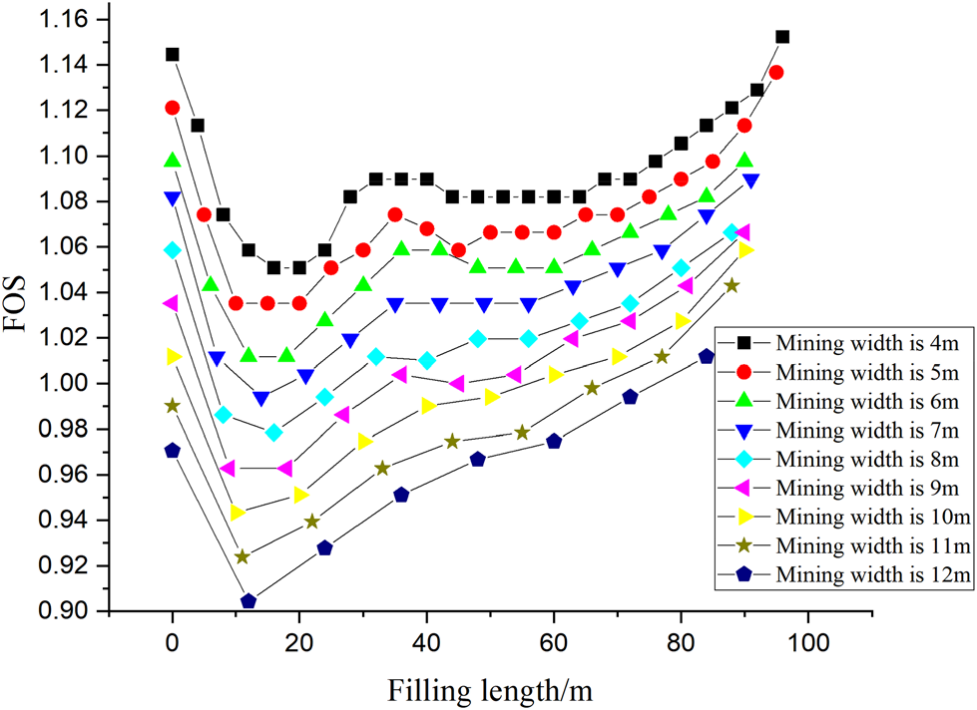
FOS of numerical simulation model with different mining width and filling length.

Based on the mining and backfill process, 125 numerical simulations were conducted, and the corresponding FOS was calculated, which can be illustrated in Fig. 4.

From analysis of the numerical simulation results, it can be found that with the increasing of mining width, the FOS of slope decrease, it can be easily understood, it is because that the suspend area under the slope increase when the mining width increase, and the slope would be more dangerous with increasing of mining width, and the corresponding FOS decrease.

**Figure 5:**
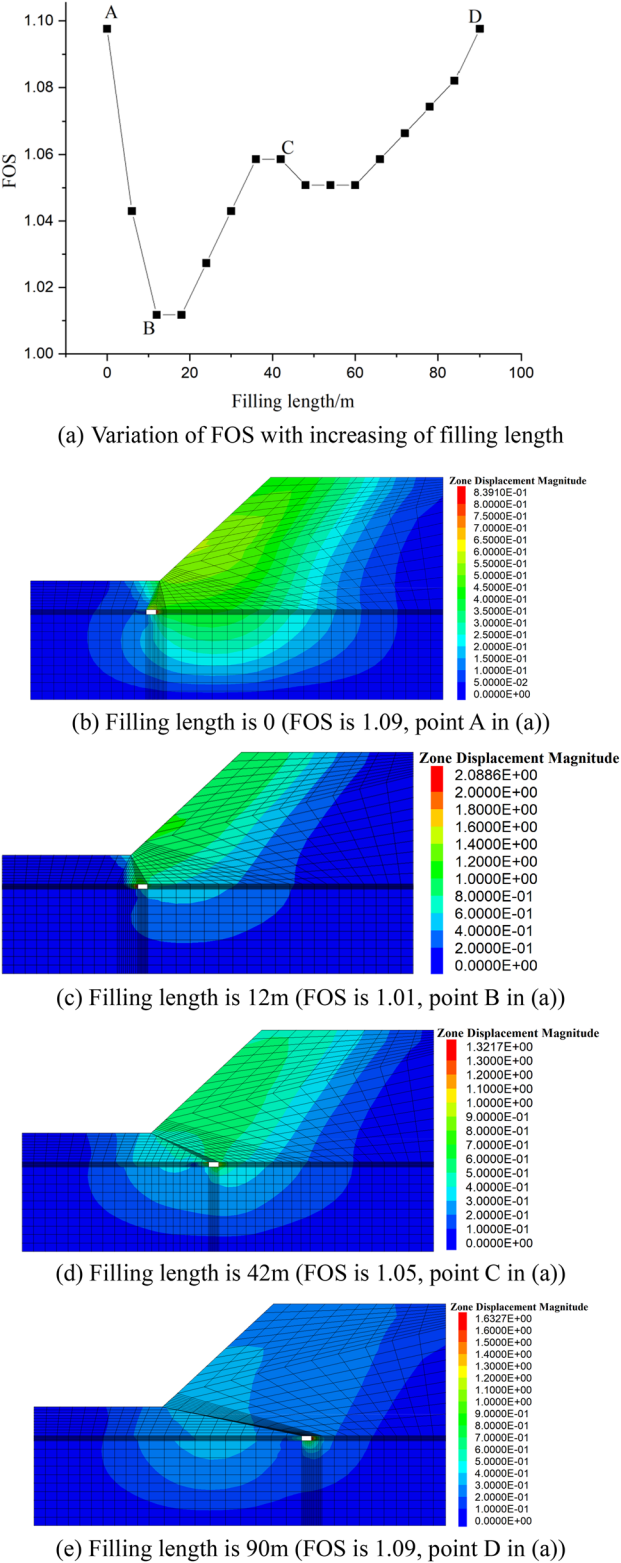
FOS and displacement magnitude contour evolution with increasing of filling length (mining width is 6 m).

Moreover, with increasing of filling length the FOS of slope decrease and then increase, taken the case with mining width 6m as example, which is displayed in Fig. 5.

It can be clearly found that with increasing of fill length (filling length is larger than 20 m), the influence of goaf to the slope stability is become more and more less, especially when the filling length is 90 m, it is can be clearly found the slope stability is little influenced by the goaf.

**Figure 6:**
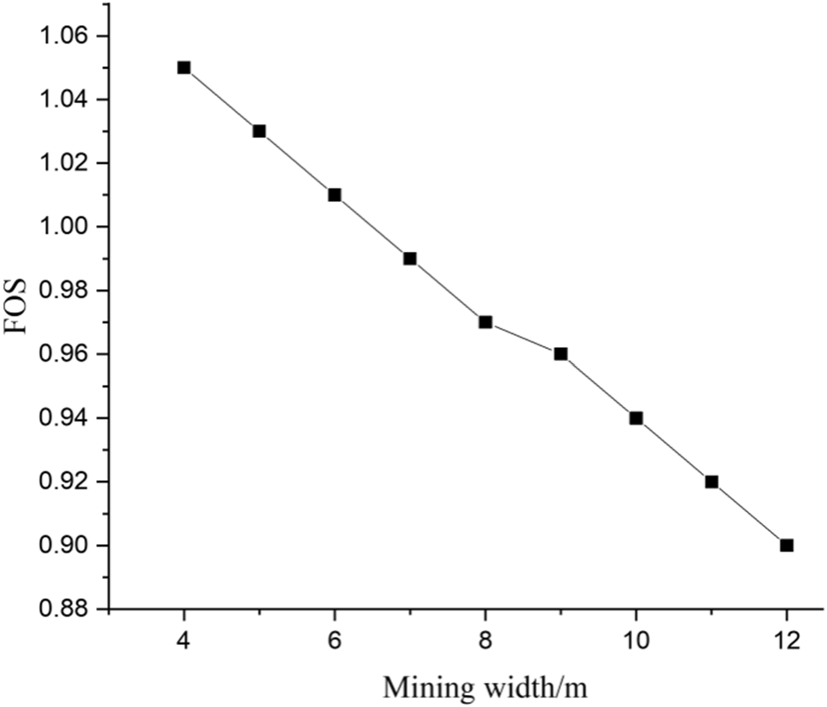
Minimum FOS with variation of mining width.

**Figure 7:**
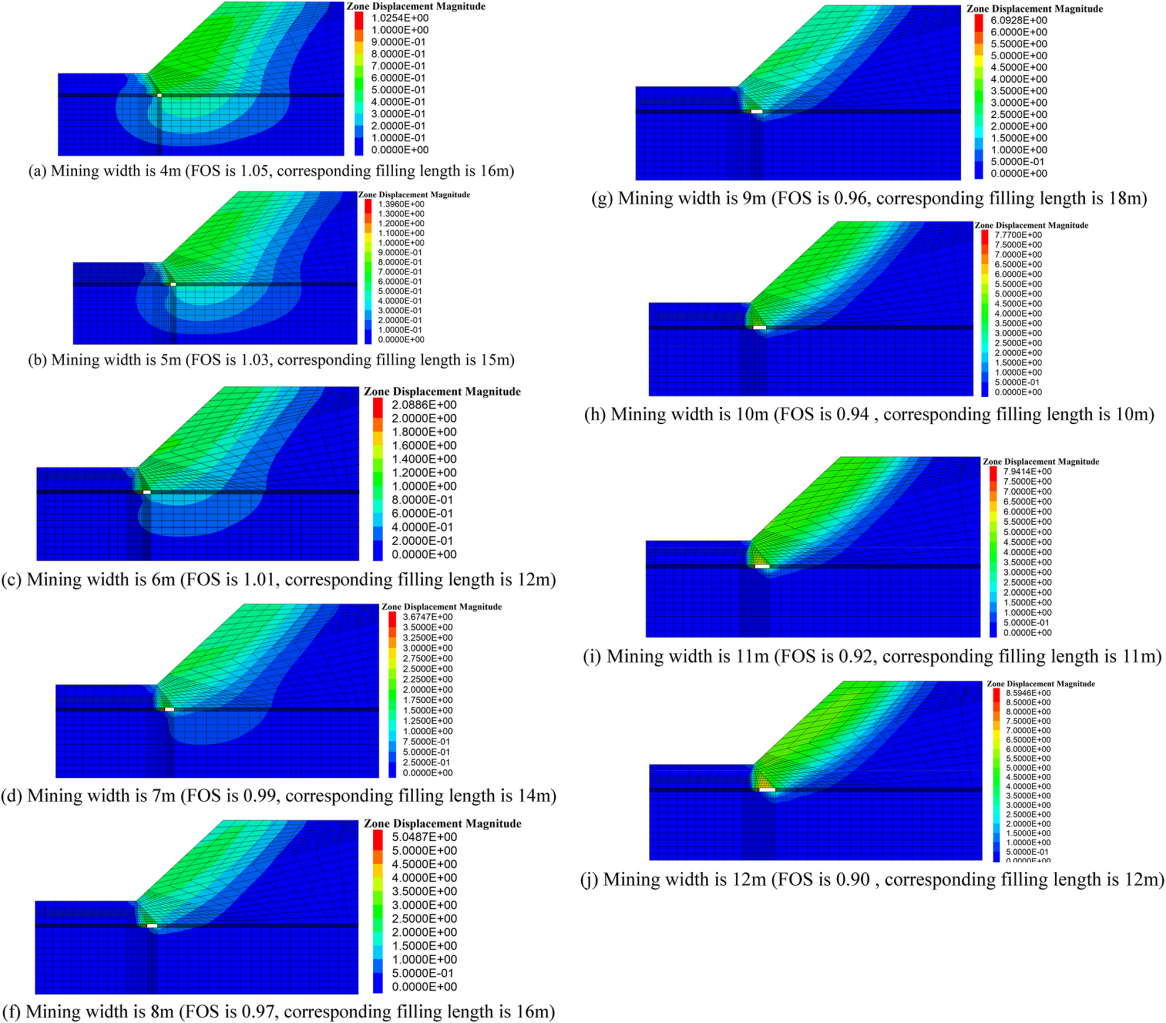
Contour of displacement magnitude.

And it should be noted that the minimum FOS is happened when the filling length falling within the ranges of 10–20 m, and the suspend area is under the toe of the slope, the influence of coal mining is significant to the stability of slope. To illustrate the influence of mining width to the stability of slope, the minimum FOS of slope with different mining width is given in Fig. 6, and the corresponding zone displacement contour is given in Fig. 7.

It can be clearly observed that the minimum FOS decrease with increasing of mining width, as well as, the FOS of slope is larger than 1 when mining width is less than or equal to 6m, indicating that the mining and backfill process is stable when the the mining width is larger than and equal to 6m. Considered the stability of slope and the mining economy, the mining width is 6m.

### Field monitoring

**Figure 8:**
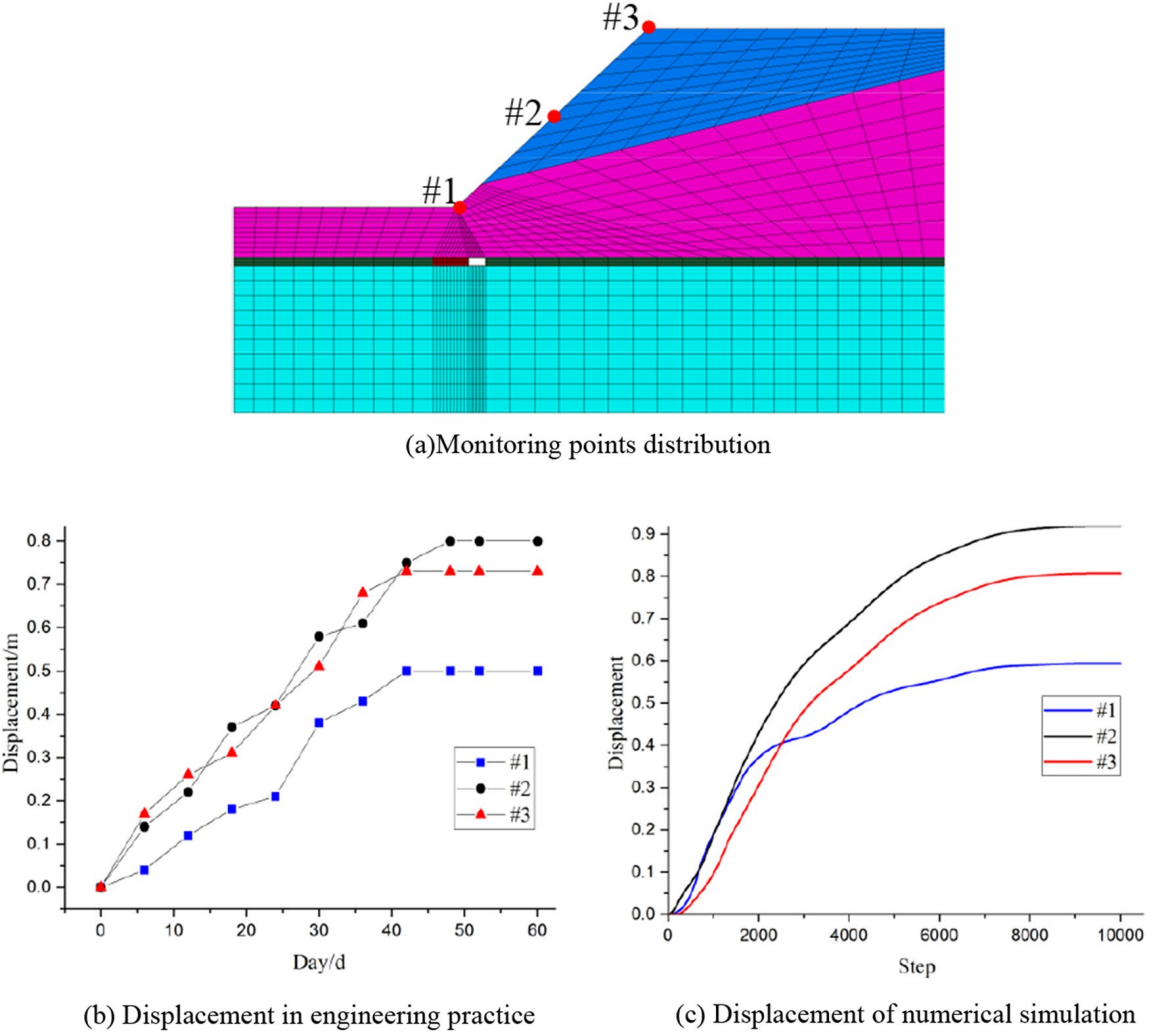
Monitoring results.

Based on the numerical simulation results, the mining width (6m) is adopted in the mining and backfill process. To guarantee the stability of mining process, and displacement of most dangerous situation (filling length is 12m (Fig. 4)) is monitored, some monitor points were distributed in the numerical simulation and the engineering practice (Fig. 8a), it can be observed that the monitoring point #1 is located at the toe of slope, and the point #3 is on the top of slope, and the point #2 is the midpoint of #1 and #3. And the monitoring results were displayed in Fig. 8b, c.

It can be found that the displacement of midpoint #2 is the largest, and the point #1 at the toe of slope is the smallest. Meanwhile, it can be noted that the displacement from the engineering practice is less than the numerical simulation, it is because that some displacement happened before, and 2 goafs were excavated and filled with backfill materials in engineering practice. Moreover, the displacement from numerical simulation and engineering practice is close, which indicated that the numerical simulation is reliable.

## Discussion

Coal is the significant strategic resource of our country, and it plays a critical important role in the development of our national economy. With the excavation of coal, more and more coal resources were discovered, and some coal was under the slope, during coal mining process, the stability of slope would be influenced, which would result in sliding of slope or collapse, thereof, the stability of slope during the coal mining process should be taken enough attention.

From analysis of the references, most studies are mainly focused on the method for estimating the stability of slope, such as finite element method, DEM, DDA, FLAC and so on. However, as for the study on the influence of coal mining to the stability of slope are less.

In this manuscript, the influence of underground coal mining to the stability of slope was analyzed by using the FLAC3D commercial software. In engineering practice, when the mining width is large, it is economy for mining production, while, the stability of slope would be decreased. Considered the stability of slope and the economy of mining production, the numerical simulation with different mining width were conducted (125 numerical simulations in total). And the FOS of slope when the mining width is different was calculated, through analysis of the numerical simulation results, it can be concluded that the FOS of slope decrease firstly and then increase with increasing of filling length, and the minimum FOS decrease with increasing of mining width. Finally, the optimal mining width is 6m, the numerical simulation result was applied to the engineering practice, based on the analysis of displacement from numerical simulation and engineering practice, it can be concluded that the numerical simulation is reliable and workable. The study provide numerical basis for the slope stability analysis.

However, it should be noted that many other factors were not considered in the numerical simulation, such as underground water, dynamic forces, and so on. These factors would reduce the stability of slope, and it would be our next task.

## Conclusions

To determine the optimal mining width, in this paper, the FLAC3D numerical simulations were conducted, and the FOS of slope was estimated, the main conclusions of this paper can be summarized as following.

1. Based on the mining process and backfill process, 125 numerical simulations in total were conducted, and the corresponding FOS of slope were calculated. It can be found that the FOS decrease and then increase with increasing of filling length, however, the minimum FOS decrease with increasing of mining width.
2. Based on the numerical simulation, the optimal mining width is set to 6 m, it is because that the FOS of slope is larger than or equal to 1 during the mining and backfill process. And the numerical simulation result is applied to the engineering practice, by comparison with the numerical simulation results and engineering practice, it can be concluded that the numerical simulation is reliable and workable.

## Data Availability

The datasets used and/or analysed during the current study available from the corresponding author on reasonable request.
